# Asymptomatic Elevation of Pancreatic Enzymes: A Case of Gullo’s Syndrome

**DOI:** 10.7759/cureus.44665

**Published:** 2023-09-04

**Authors:** Guilherme Camões, Diana M Ferreira, Arsénio Santos, Lèlita Santos

**Affiliations:** 1 Internal Medicine, Centro Hospitalar e Universitário de Coimbra, Coimbra, PRT

**Keywords:** gastroenterology, pancreatic enzymes, pancreas, benign pancreatic hyperenzymemia, gullo’s syndrome

## Abstract

Benign pancreatic hyperenzymemia (BPH) or Gullo’s syndrome is a benign condition consisting of an oscillating elevation of pancreatic enzymes without the identification of pancreatic disease. Its diagnosis is usually incidental and by excluding other conditions that occur with elevated pancreatic enzymes. To the best of our knowledge, there are no reports of this diagnosis to this day in Portugal.

A 65-year-old female was referred to an internal medicine consultation for complaints of xerostomia, xerophthalmia, and xeroderma with one year of evolution. From the study carried out by the patient prior to the consultation, an incidental elevation of amylase stands out. The sicca symptoms were attributed to sertraline since, after excluding other causes, its discontinuation resolved the symptoms. Regarding the elevation of pancreatic enzymes, the patient underwent an extensive diagnostic study for clarification without identifying any condition. The serial measurement of amylase and lipase revealed an oscillating increase in pancreatic enzymes with temporary normalization. After one year of follow-up, the diagnosis of Gullo’s syndrome was established.

The identification of Gullo’s syndrome is extremely important as it avoids carrying out unnecessary tests in the future and allows the patient to be reassured in the face of this benign alteration of pancreatic enzymes. A follow-up of at least one year is crucial since some pancreatic tumors course with an asymptomatic increase in pancreatic enzymes.

## Introduction

Benign pancreatic hyperenzymemia (BPH) or Gullo’s syndrome consists of an asymptomatic persistent elevation of pancreatic enzymes (amylase, pancreatic isoamylase, lipase, and trypsin) without the identification of any pancreatic disease [1-8]. This disorder was first described by Gullo in 1996, and since then, it has also been called Gullo’s syndrome [7]. This diagnosis is usually incidental [1,2,4,8] and is made by exclusion of pancreatic and non-pancreatic diseases that lead to the elevation of pancreatic enzymes. Although pancreatic enzymes are usually elevated, they can return to normality temporarily [1-6,8]. We present a case of BPH to highlight the benign nature of this condition and the importance of its identification to avoid unnecessary diagnostic examinations and decrease patient anxiety.

## Case presentation

A 65-year-old female was referred to internal medicine consultation by her general physician (GP) with complaints of xerostomia, xerophthalmia, and xeroderma with one year of evolution. She also reported weight loss (5 kg in six months) and dysphagia conditioned by xerostomia.

The patient’s medical history included diabetes mellitus type 2, hyperuricemia with gout, osteoporosis, depression, initial insomnia, and active smoking (seven pack-years). Her home medications included sitagliptin + metformin 50/1,000 mg, sertraline 50 mg, alprazolam 1 mg, trazodone 100 mg, calcium carbonate 1,500 mg, and artificial tears.

Vital signs on the appointment showed a temperature of 36.2°C, blood pressure of 140/70 mmHg, pulse rate of 65 per minute, and respiratory rate of 16 per minute. Her weight was 52 kg, and her height was 1.60 m, corresponding to a body mass index of 20.3 kg/m^2^. Her physical examination was unremarkable.

The patient brought some diagnostic tests prescribed by the GP, namely, an ultrasound of the salivary glands without abnormalities and a blood work that highlighted an increase in amylase.

To ascertain the etiology of the sicca symptoms, the patient underwent several diagnostic tests, including a Schirmer’s test that proved to be normal, an autoimmunity study that did not reveal the presence of anti-SS-A/Ro autoantibodies, and a scintigram of the salivary glands that revealed normal function. Since she did not meet the classification criteria for Sjogren’s syndrome, we suspected an adverse reaction to antidepressants. The patient suspended sertraline with a resolution of sicca symptoms.

**Figure 1:**
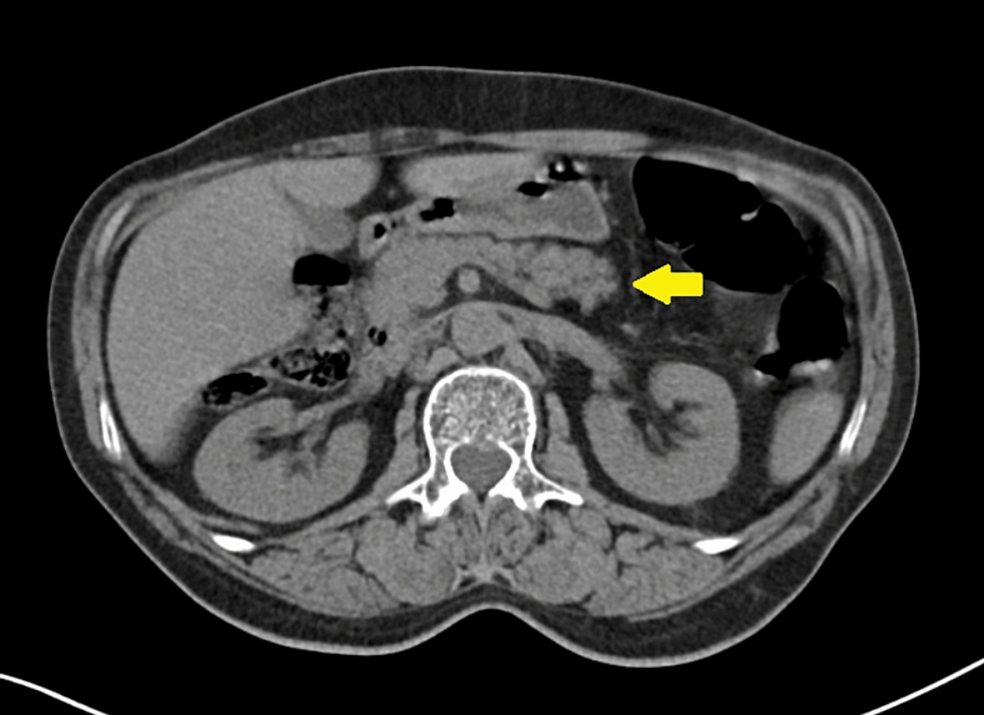
Abdominal computed tomography without contrast demonstrating pancreas without abnormalities (yellow arrow).

**Figure 2:**
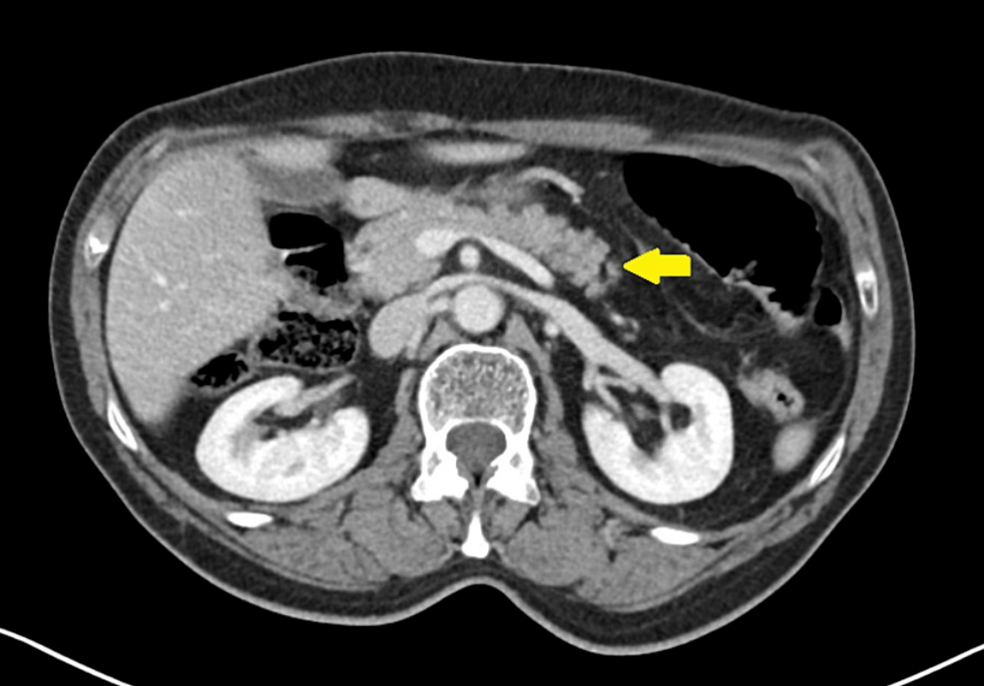
Abdominal computed tomography with contrast demonstrating pancreas without abnormalities (yellow arrow).

Regarding the elevation of amylase, an incidental finding of the analytical study requested by the GP, we started the diagnostic pathway by carrying out a new analytical study with serum amylase and lipase measurement, urinary amylase measurement, and upper abdominal ultrasound. Serum measurements of amylase and lipase were elevated (amylase: 157 U/L (normal range: 25-125U/L), lipase: 125 U/L (normal value: <67 U/L)), urinary amylase was normal (160 U/L) (normal range: 42-321 U/L), and ultrasound did not reveal abnormalities. Given the maintenance of elevated amylase and this being accompanied by elevated lipase, the patient underwent an abdominal computed tomography scan to exclude pancreatic and non-pancreatic conditions that justify this elevation. This examination did not reveal any abnormalities (Figures 1, 2).

**Figure 3:**
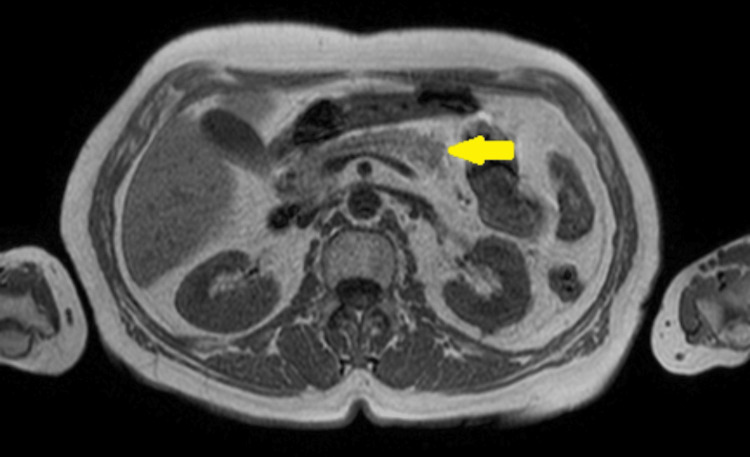
Pancreatic nuclear magnetic resonance in T1 sequence demonstrating pancreas without abnormalities (yellow arrow).

**Figure 4:**
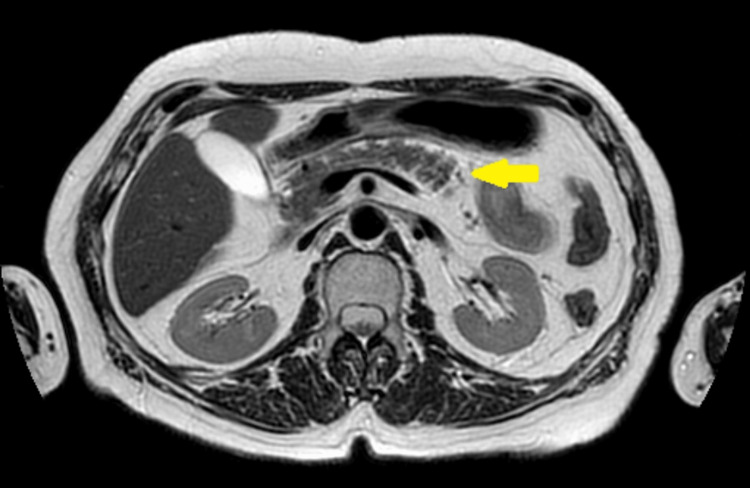
Pancreatic nuclear magnetic resonance in T2 sequence demonstrating pancreas without abnormalities (yellow arrow).

**Figure 5:**
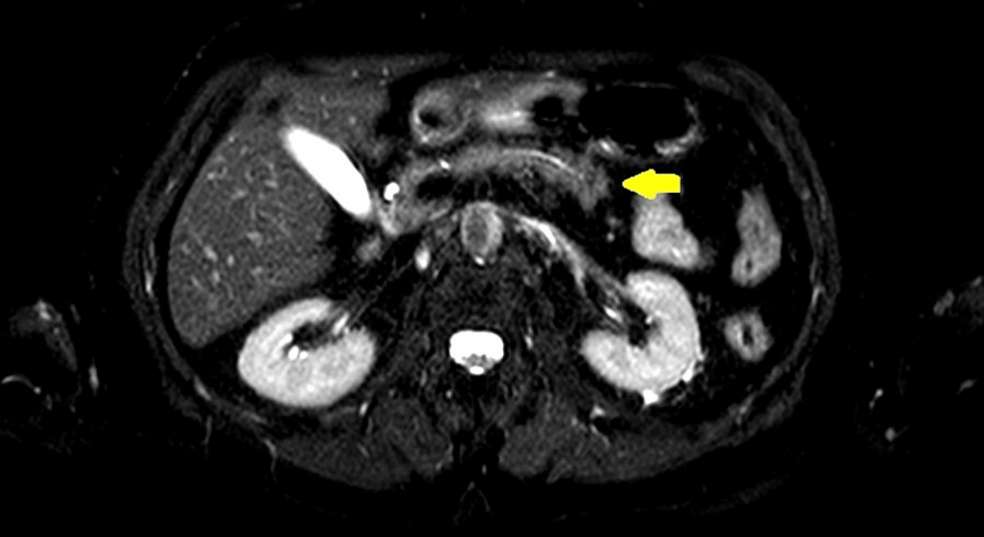
Pancreatic nuclear magnetic resonance in T2 SPAIR sequence demonstrating pancreas without abnormalities (yellow arrow). SPAIR: spectral attenuated inversion recovery

An abdominal nuclear magnetic resonance was then requested for further clarification, which also did not reveal any alteration (Figures 3-5).

Over the time that passed between consultations and the performance of imaging examinations, the patient was also carrying out amylase and lipase measurements, and we found that the values fluctuated and some reached normality (with the higher values being 189 U/L for amylase and 193 U/L for lipase). Bearing in mind the previous information, Gullo’s syndrome was suspected, and it was proposed to carry out serial fasting measurements for five consecutive days to support this diagnosis. The values obtained are represented in Table 1.

**Table 1:** Serial measurement of serum amylase (normal range: 25-125 U/L) and lipase (normal value: <67 U/L).

Day	1	2	3	4	5
Amylase (U/L)	88	131	85	80	75
Lipase (U/L)	39	109	35	37	35

After analyses of the measurements, we confirmed the diagnosis of Gullo’s syndrome, since there is daily oscillation of the amylase and lipase values and a follow-up for at least one year without any pathology identified. Given the benignity of this condition, no treatment was instituted, and the patient was followed up for two years without developing any symptoms.

## Discussion

Elevation of pancreatic enzymes could represent an underlying pancreatic condition. However, in BPH, oscillating elevation of pancreatic enzymes with transitory normalization without identification of any pancreatic condition is the rule [1-6,8]. The incidence is unknown [8]. This affects more males than females with a ratio of 1.5:1 [1,6]. It can occur sporadically or hereditary and affects persons of all ages [1-6,8]. In most cases, all pancreatic enzymes are elevated (90%-95%), but in 5%-10% of cases, only amylase or lipase is elevated [2,6]. The elevation is usually in the range of 1.5-6 times the upper normal limit, but it can be higher, up to 15 times, and lipase is typically more elevated [2,4,8]. Discriminating the variations of pancreatic enzymes individually, Gullo [7] reports in his study an increase above the upper normal limit in the range of 1.4-4.1-fold for amylase, 1.8-6-fold for pancreatic isoamylase, 1.5-7.7-fold for lipase, and 1.6-13.9-fold for trypsin. Regarding daily fluctuations, Gullo [9] carried out a study in which 42 patients diagnosed with BPH underwent serial measurements of pancreatic enzymes for five consecutive days. The study reports that 19% of patients present fluctuations without reaching normalization, 78.6% present fluctuations reaching normalization, and all enzymes were normal in 2.4%. The number of normalizations varies between 1 and 4 determinations. Of the 41 patients who presented hyperenzymemia, 37 showed an increase in all enzymes, with lipase and trypsin having the highest values, three showed an increase only in amylase and pancreatic isoamylase, and only one in lipase. The pathogenesis remains unknown. Nevertheless, there are some hypotheses that try to explain this condition, such as a cellular defect of acinar cells leading to pancreatic enzyme outflow into the blood circulation [1-6], the effect of secretin in the pancreatic duct of Wirsung [1,2,4], and the dysfunction of sphincter of Oddi not sufficient to cause symptomatology or pancreatic steatosis [3]. An association between serine protease inhibitor Kazal-type 1 (SPINK1), serine protease 1 (PRSS1), and cystic fibrosis transmembrane conductance regulator (CFTR) mutations and BPH was not found [4,10]. There are currently no known associated conditions [5]. For the diagnosis of BPH, oscillating elevation of pancreatic enzymes with temporary normalization, at least one year of follow-up, and exclusion of pancreatic conditions are required [1-6,8]. A follow-up of at least one year is important since 1%-2% of pancreatic cancers, especially in the elderly, present with asymptomatic elevation of pancreatic enzymes [1,2,4,6].

Similar cases have been reported. Yang et al. [11] reported a 57-year-old Chinese male who presented with an elevation of pancreatic enzymes after undergoing checkup tests. The patient was asymptomatic and denied a family history of known pancreatic pathology or consumption of alcohol, tobacco, or hepatotoxic agents. The patient performed several diagnostic tests that revealed hyperamylasemia (amylase: 330 U/L (normal range: 28-100 U/L)) and hyperlipasemia (lipase: 619 U/L (normal range: 22-51 U/L)). Subsequently, he performed several imaging tests, including abdominal ultrasound, abdominal computed tomography, and magnetic retrograde cholangiopancreatography. All revealed normal findings. The patient remained under follow-up, including regular amylase and lipase measurements. After two years of follow-up, the patient was diagnosed with BPH after remaining asymptomatic and without pancreatic structural changes.

## Conclusions

Benign pancreatic hyperenzymemia is a benign condition, and its identification is important to avoid unnecessary tests and reassure the patient. Follow-up is essential to determine the benignity of the elevation of pancreatic enzymes, given the possibility of being an early manifestation of pancreatic tumors. To the best of our knowledge, this is the first case reported in Portugal.
